# External-forcing modulation on temporal variations of hydrothermalism-evidence from sediment cores in a submarine venting field off northeastern Taiwan

**DOI:** 10.1371/journal.pone.0207774

**Published:** 2018-11-29

**Authors:** Jia-Jang Hung, Hsiang-Yi Yeh, Shao-Hung Peng, Yuan-Pin Chang, Jiang-Shiou Hwang

**Affiliations:** 1 Department of Oceanography, Asian-Pacific Ocean Research Center, National Sun Yat-sen University, Kaohsiung, Taiwan; 2 Institute of Marine Biology, National Taiwan Ocean University, Keelung, Taiwan; KAOHSIUNG MEDICAL UNIVERSITY, TAIWAN

## Abstract

The temporal variation of sulfur and metals in core sediments off Kueishantao Islet, a hydrothermal vent site at northeastern Taiwan, was explored to elucidate the changes in submarine hydrothermal emanation over a centennial time scale. The discharge of acidic fluids containing abundant sulfides and dissolved metals results in different concentrations of sulfur and metal accumulating in deposited sediments. In addition to particle size and organic carbon affecting metal contents, the content of total sulfur (TS), which is regarded as an indicator of hydrothermalism, correlates positively and strongly with Fe and other metals; however, it correlates negatively with another index of hydrothermalism, the Al/(Al+Fe+Mn) ratio. The TS content in Core Ks2, the core closest to the vents, increased during 1950–1956, 1968–1970, 1982–1987, 1990–1992, and 2004–2005, but decreased during 1967–1968, 1988–1990, and 1994–1995. The chronological changes in the TS concentration of Cores Ks3 and S2 were very similar to those of Core Ks2 within the aforementioned time spans. The numerous large earthquakes (M_L_ > 5) and typhoons that affect northeastern Taiwan appear to influence hydrothermal emanation and determine the temporal variation of sulfur and metals in sediment cores.

## Introduction

Submarine hydrothermalism has recently drawn a substantial attention because it occurs generally in the shallow regimes of global ocean and releases enriched dissolved substances to the upper layer of water columns. Those emanated substances may be either beneficial or harmful to the shallow marine ecosystem. Because the shallow hydrothermal ecosystem is generally maintained by organic carbon synthesis and energy flow driven by both photosynthetic and chemosynthetic processes, the biological community composition can be quite different from that found in the deep-sea hydrothermal ecosystem [1, 2]. It is generally consensus that the biological communities are more diverse and unique in shallow than in deep hydrothermal ecosystems [1, 2]. However, the shallow hydrothermal field is also subject to atmospheric and anthropogenic impacts and its physical environment, habitat and associated biological communities can be often changed spatially and temporally.

Located at the western end of the southernmost part of the Okinawa Trough and 10 km away from the northeastern coast of Taiwan, the offshore Kueishantao (KST) Islet is a small active volcanic island. The last volcanic eruption was reported to occur sometime 7 ka before the presence [3, 4]. Very high magmatic activity was suspected to occur beneath KST Islet because of high ^3^He–^4^He ratios in bubble-gas samples implying the mantle as the main source of gases [5]. Previous studies found that most hydrothermal vents locate on the eastern side off the KST Islet at a depth range of 10 − 30 m. The hydrothermal vents which discharge yellow and white colors of fluids and plumes are generally regarded as yellow vents and white vents, respectively [6]. Previous studies also revealed that the ejected fluids and plumes were quite hot and acidic; the highest temperature and lowest pH were recorded at 116°C and 1.52, respectively [6–7]. Very high acidic fluids may result from high concentrations of CO_2_ gas (>92%) and small quantities of H_2_S (0.8–8.4%) [6, 9]. The influence of discharged fluids on the water column and sediments in the hydrothermal field is not limited to the venting zone because of strong impacts of tidal and Kuroshio currents in the area [6, 7].

Our previous study has shown very high concentrations of dissolved HS^-^ and metals in KST venting fluids in spite of high temporal variability [10]. The concentration was much higher in fluids than the surrounding seawater possibly because of very acidic fluids in leaching and mobilizing metals from the underlying rocks and sediments [10]. In the first study of KST venting systems, Chen et al. [6, 8] also found a dozen of enriched metals in vent fluids and the concentrations of some metals (Si, Fe and Mn) were 2−6 orders of magnitude higher than the background seawater levels. Many other submarine hydrothermal vents around the world also emanated hydrothermal fluids containing enriched dissolved sulfide and metals. For instance, very high concentrations of dissolved B, Fe, As, Sb, Mn, Si, and Li were reported in the hydrothermal fluid of Champagne Hot Springs (CHS), Dominica, Lesser Antilles, likely resulted from hydrothermal leaching of sand patches and little mixing with seawater [11]. The process of phase separation may be responsible for the enrichment of dissolved As in hydrothermal fluids of Milos Island in Mediterranean Sea and Tutum Bay near the Ambitle Island [12–14]. Cardigos et al. [15] also found increased concentrations of dissolved Ba, Fe and Mn, Pb in yellow-zone fluids and dissolved Pb in white-zone fluids derived from D. João de Castro seamount (Azores). A pronounced discharge of dissolved sulfide and certain metals from Excess Si, alkalinity, Ca^2+^, Sr^2+^, and Mn in seawater are possibly effects of groundwater or the dissolution or hydrothermal alteration of rockssubmarine warm springs in Italy was likely caused from leaching volcano sands by acidic fluids [16]. The elevated concentrations of dissolved sulfide and metals in fluids were likely derived either from phase separation or from leaching of rocks and sediments by acidic fluids. Also the metal mobility in fluids and plumes may be significantly enhanced by binding of organic compounds to metals [15, 17]. Those dissolved sulfide and metals derived from venting fluids may precipitate on seafloors or incorporate into in-situ or remote sediments through various geochemical processes [18–20]. In the KST venting field, Hung et al. [10] observed a conspicuous influence of hydrothermalism on the spatial distribution of sulfides and metals in surface sediments.

Although the effects of submarine hydrothermalism on spatial distributions of metals in surface sediments have been widely documented as shown in the preceding references, rather limited data are available on the temporal variation of sulfides and metal contents. This holds particularly to their relationship with historical changes in hydrothermal activity if very short-term investigations through yearly sampling are excluded. Previous studies were able to detect recent changes in venting activity in the study area [6, 7]. Our recent aerial photos revealed a marked change in the sulfide spreading zone between 2012 and 2016 (S1 Fig). Because no long-term observations are available, it is unclear whether this change was associated with short-term or long-term eruption periodicity. In addition, our recent investigation through diving also found a pronounced change of venting sites after a local earthquake. These recent findings motivated us to examine the intermediate effects of emission on the variability of TS and metals in dated sediments. However, the concentration of a metal in sediments is determined not only by its source but also by the texture (particle size) and organic content of the sediments [21, 22]. Thus, it is necessary to understand the overlapping influence of physical–geochemical properties and hydrothermalism before evaluating the effects of temporal changes in hydrothermal activity on sulfide and metal accumulation in sediments. As the KST Islet locates on the hot spot of seismic activity and typhoon pathway, we propose a hypothesis that the temporal variability of hydrothermal discharge is dependent mainly on the impact of external forcings on the KST venting field.

## Materials and methods

Three sediment cores (Core Ks2, Core Ks3, and Core S2), which were close to vents and successfully dated, were selected for the study. The cores were collected using gravity and piston corers onboard the Ocean Researcher II in April and September 2009. The core thickness was approximately 88 cm, 80 cm and 90 cm for Core Ks2, Ks3 and S2, respectively. In order to verify the source of sulfide and metals in sediments derived mainly from hydrothermal vents, the fluid and plume samples (n > 50) were collected from yellow and white vents to determine the concentration of dissolved sulfide and metals. The sampling was performed by SCUBA divers by using a home-made titanium tube equipped with a temperature sensor, a controllable valve and an air-tight Pyrex glass bottle. The air pressure of glass bottle was reduced prior to taking sample in order to draw the hydrothermal solution quickly into the bottle. The hydrothermal fluids and plumes were taken from the interior of vent orifices and various positions of buoyant plumes, respectively. pH was measured on a boat after collection with a portable pH meter (Mettler Toledo MP 120, Germany). The precision was estimated to be 0.01 pH unit. In addition, the background seawater samples (n = 9) were collected from remote Kuroshio Current for comparing the levels of dissolved sulfide and metals in seawater, fluids and plumes. All sampling tools and bottles were acid cleaned and thoroughly rinsed with distilled deionized water (DDW) before sampling to avoid any contamination.

**Fig 1:**
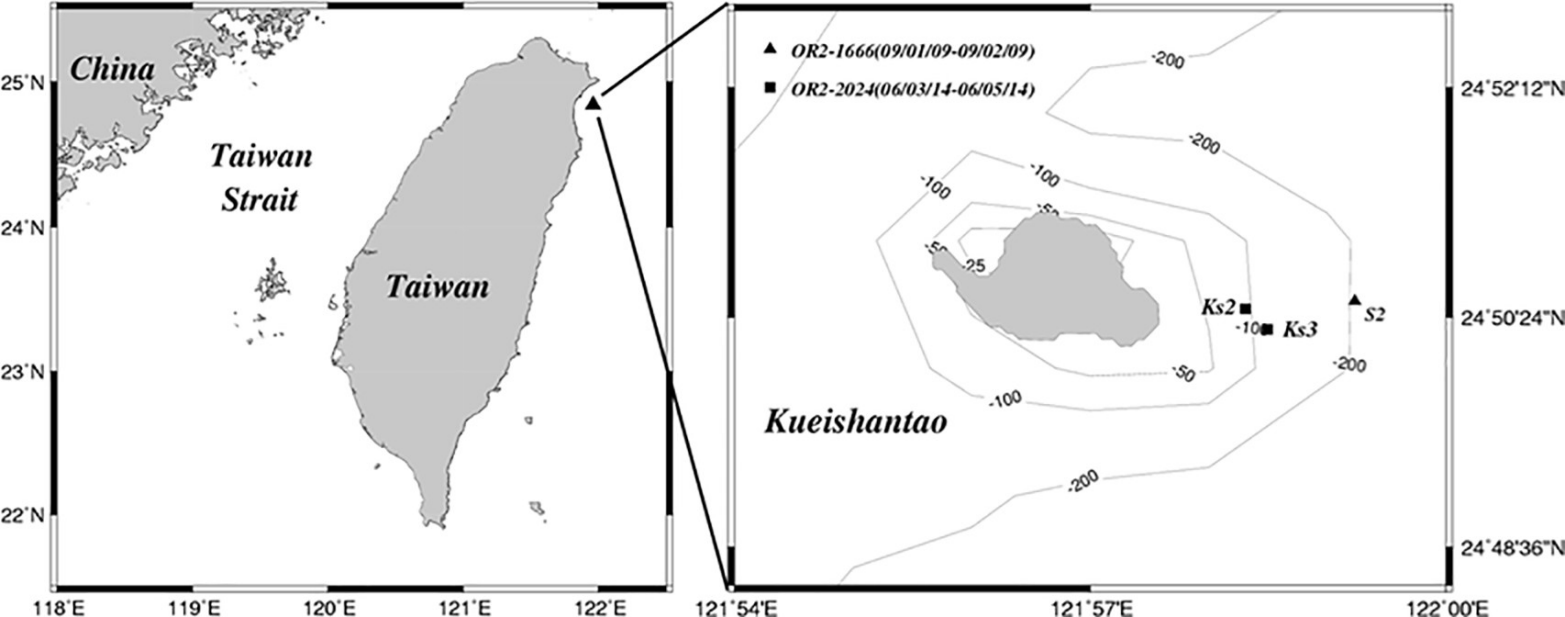
Study area and locations of three cores in the Kueishantao venting field off northeastern Taiwan.

Dissolved sulfide in the collected samples was determined with the methylene blue method [23] immediately after the samples were shipped back to the land laboratory. The precision was generally better than 0.7% (n = 6) based on a concentration of 20 μM HS^−^ in artificial seawater. Determination of dissolved metals were carried out in a clean room by using acid-washed POLYCAP cartridges (0.2 μm pore size, Whatman, USA) for online filtration under a closed condition. After filtration, the seawater was preconcentrated with commercial chelating column (Dionex MetPac CC-1, USA) widely used for concentrating transition metals in seawater. The preconcentrated metals were determined by inductively coupled plasma mass spectrometer (ICPMS, Element XR, Thermo, USA). Precision and accuracy were generally better than 5% by determining the reference samples of NASS-5 and CASS-4 (NRC, Canada). Detailed procedures in metal preconcentration and determination can be referred to Ho et al. [24]. The sampling locations for three sediment cores are shown in Fig 1. The depth of core location was approximately 60 m in Core Ks2 and Core Ks3 and 180 m in Core S2. The distance was about 500 m between Core Ks2 and Core Ks3, and about 1.8 km between Core Ks2 and Core S2. The retrieved cores were placed on a hydraulic jack to eject the sediments out of core liners, and the sediments of each core were then sliced into 2-cm intervals. The individual sediment samples were frozen before being taken to a land laboratory for further treatment.

Each sediment sample was gently squeezed to break up its aggregates and sifted through a nylon sieve to remove particles bigger than 1 mm. A part of screened sediment was used for particle-size determination with a particle-size counter (Beckman Coulter LS Particle Size Analyzer, USA). The clay and mud contents were estimated and reported for particle fractions smaller than 2 and 63 μm, respectively, in this study. To prevent sulfide oxidation before determination, the sulfur content was determined from the sediment which was freeze dried directly without washing with DDW to remove sea salt. Subsequently, the freeze dried sediment was ground to powder using an agate mortar and pestle, before undergoing analyses for contents of total sulfur (TS), organic carbon, and metals in sediments. TS was measured directly by using a C/N/S analyzer (Fisons NCS 1500, UK), whereas inorganic carbon was removed with hydrochloric acid before total organic carbon (TOC) and total nitrogen were also measured with the C/N/S analyzer [21, 22]. Both TS and TOC were analyzed in triplicates. The precision of TS and TOC determination was generally better than 2% when measuring a local estuarine sediment (n = 9) for standardization.

For analyzing the metals in duplicate sediment samples, 0.2 g portion of ground sediment was weighed and transferred into a pressure-resistant vessel, and the mixture of super-pure acids (HNO_3_:HCl:HF = 3:3:4) was added for digestion. The vessel was heated stepwise in a microwave oven (CEM 2000, USA). The acid dissolved solution was then diluted with Milli-Q water before the metals were measured. The total concentrations of metals in digested solution were determined using an inductively coupled plasma optical emission spectrometer (ICP-OES, Perkin Elmer Optima 2100DV, USA) [10, 25]. Both standards and samples were added with an internal standard (Y) throughout the measurements. Analytical precision and accuracy were evaluated by analyzing standard reference materials, NIST SRM 1646 and PACS-1 (SRM of the National Research Council of Canada), with the same procedures. The uncertainty (n = 9) was <5% for Cd, <4% for Cu, Ni, Zn, and <2% for Al, Cr, Fe, Mn and Zn.

The sediment chronology of each core was constructed from the sedimentation rate that was determined with ^210^Pb isotope method according to a steady-state advection-decay model. The derived chronology was verified using ^137^Cs stratigraphy, which in steady sedimentary environments typically shows a subsurface peak indicating maximum nuclear fallout at approximately 1963 AD. The ^210^Pb-based sedimentation rates in this study were mostly consistent with those estimated according to the depth of the subsurface ^137^Cs peak (indicating 1963 A.D.) and the penetration depth of ^137^Cs (approximately1950 A.D.). ^210^Pb and ^137^Cs were measured simultaneously through nondestructive gamma spectrometry, based on their photon energies of 46.52 and 661.62 keV, respectively. The activity of ^214^Pb (at 351.99 keV), a precursor of ^210^Pb, was measured and was then subtracted from the measured activity of ^210^Pb to yield the activity of unsupported ^210^Pb (also called excess ^210^Pb). Four HPGe detectors were employed in this study. The detectors were calibrated using IAEA standards 133A, 327, and 375 for a sample weight at 100 g, as a reference, coupled with an in-house working standard for various masses of 10–250 g. Detailed procedures can be found in Huh et al. [26]. The sedimentation rate (S) was derived from the slope of a plot between lnC (excess ^210^Pb) and depth (cm) (S = −λ/slope, where λ is the ^210^Pb decay constant (0.0311 yr^−1^)).

## Results

### Main source of dissolved sulfide and metals in the venting field

Given the fact that sulfide and metals in sediments are mainly derived from hydrothermal vents, the distribution of temperature, pH, dissolved sulfide and metals in hydrothermal fluids, plumes and Kuroshio seawater was presented in Table 1 for comparison. The very high temperature (up to 112°C) and concentrations of dissolved sulfide (147−2227 μM) and dissolved metals, as well as the low pH (lowest at 1.52) of the KST fluids and plumes indicate that a substantial amount of dissolved sulfide and metals originated from hot and acidic hydrothermal vents. The highest concentrations of dissolved sulfide and metals in KST fluids and plumes were several orders of magnitude higher than those in the background Kuroshio seawater. When compared with other submarine hydrothermal systems [11, 12, 27], they were slightly higher or in the same orders for dissolved sulfide and various dissolved metals. Notably, total discharge was much higher for Al, Fe, and Mn than for other metals, but hydrothermal Al may not significantly increase the concentration of Al in sediments because of the particularly high concentration of Al in marine sediments. The magnitude of hydrothermal metal fluxes may not be comparable with those from deep-sea hydrothermal vents (e.g. 21°N, East Pacific Rise) [28], but these fluxes may significantly elevate metal concentrations in sediments in a small shallow hydrothermal field.

**Table 1:** A list of mean and ranging concentrations of dissolved metals in hydrothermal fluids and plumes and associated offshore Kuroshio seawater.

Type	Conc.	Temp (°C)	pH	Al (μM)	Cd (nM)	Co (nM)	Cr (nM)	Cu (nM)	Fe (μM)	Mn (μM)	Ni (nM)	Pb (nM)	Zn (nM)
**Fluid**	**Ave.**	83.7	3.38	92.0	1.75	1.94	106	135	6.44	2.07	14.6	12.2	361.2
**and**	**Sd**	27.5	1.60	71.5	1.99	2.90	83.5	172	4.70	3.74	15.5	17.1	661.2
**plume**	**Max**	112	6.38	210	5.37	10.3	221	408	17.6	12.8	54.9	54.7	2321
	**Min**	52	1.76	9.94	0.14	0.12	8.33	20	0.92	0.14	4.82	0.80	7.0
**Discharge**				(×10^5^)	(×10)	(×10)	(×10)	(×10^3^)	(×10^4^)	(×10^4^)	(×10)	(×10)	(×10^3^)
Rate*				7.02	1.33	1.40	8.06	1.03	4.89	1.58	1.11	9.47	2.74
(mol yr^-1^)													
**Kuroshio seawater conc.**		27−28	7.9−8.1	0.08	0.05	0.83	—	9.0	0.002	0.001	1.20	0.30	5.0

*Discharge rates of metals were estimated from metal concentrations in fluid (this study) and discharge rate of fluid estimated by Chen et al. (2005) [6].

### Distributions of grain size, organic carbon, and total sulfur contents in sediments

**Fig 2:**
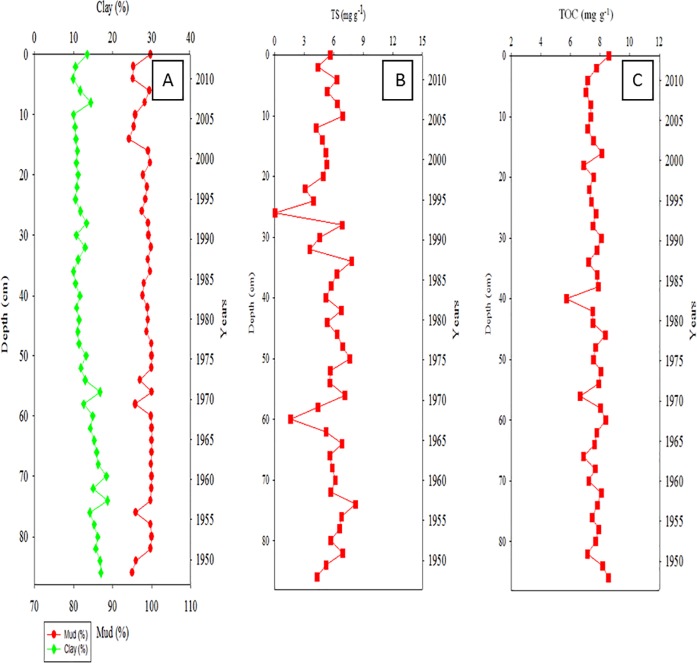
Distributions of fine particle (clay & mud, A), total sulfur (TS, B), and total organic carbon (TOC, C) in Core Ks2 sediments.

**Fig 3:**
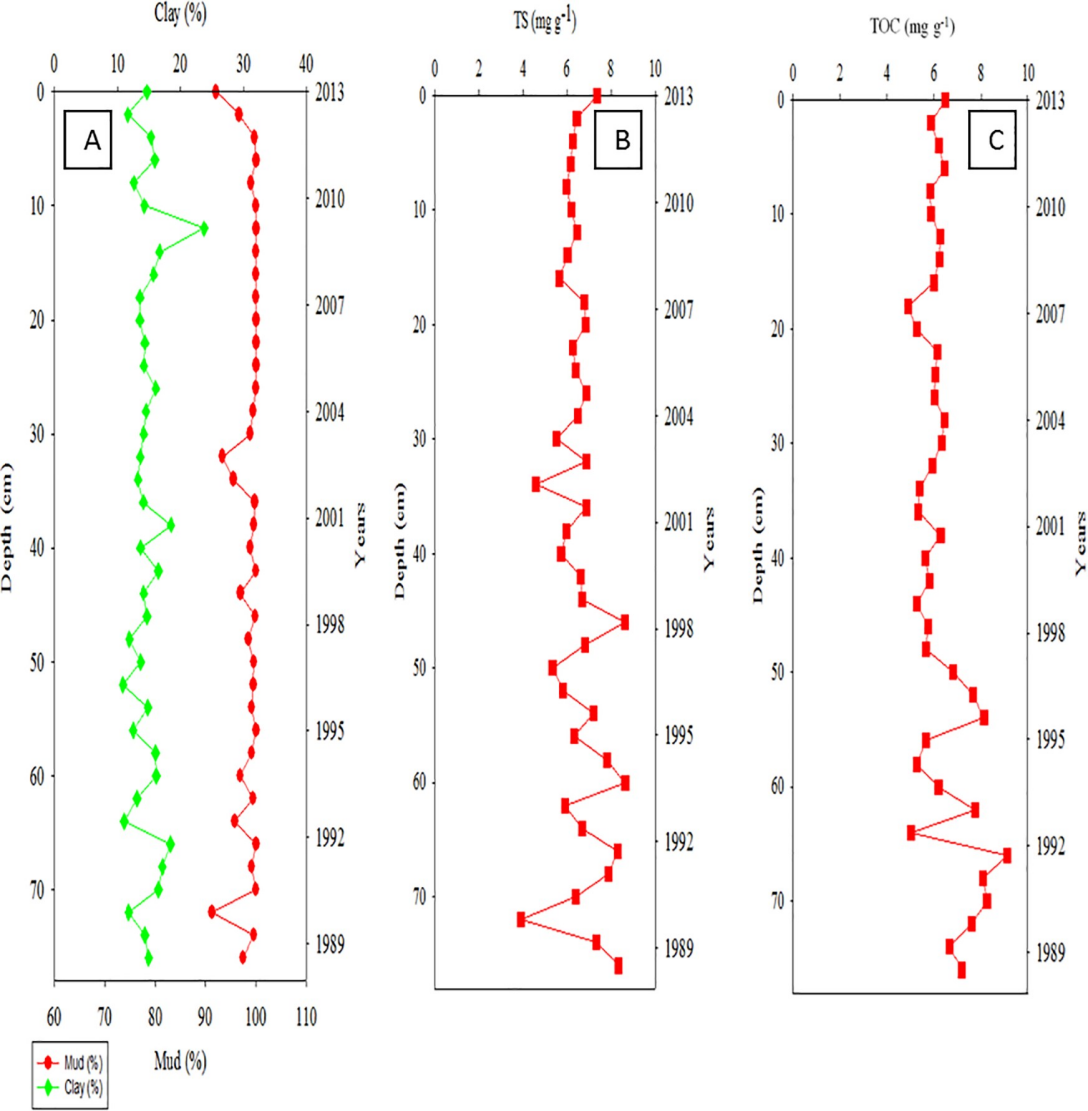
Distributions of fine particle (clay & mud, A), total sulfur (TS, B), and total carbon (TOC, C) in Core Ks3 sediments.

**Fig 4:**
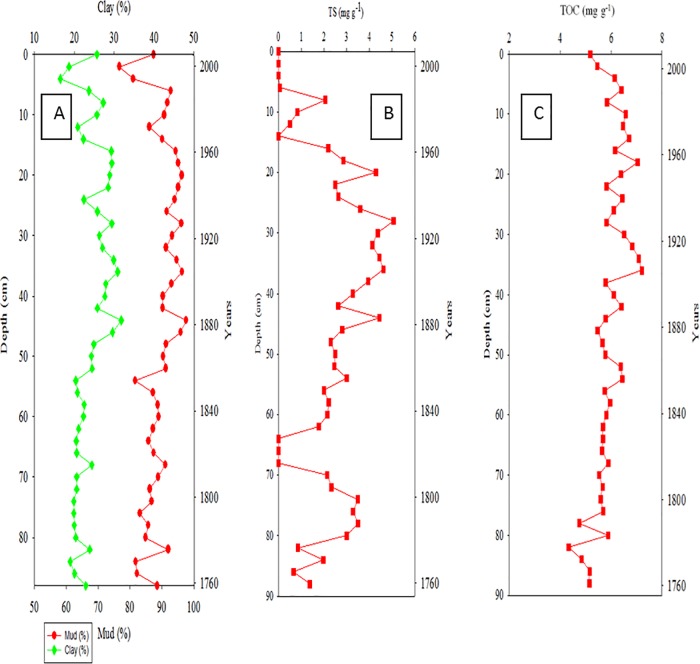
Distributions of fine particle (clay & mud, A), total sulfur (TS, B), and total organic carbon (TOC, C) in Core S2 sediments.

**Fig 5:**
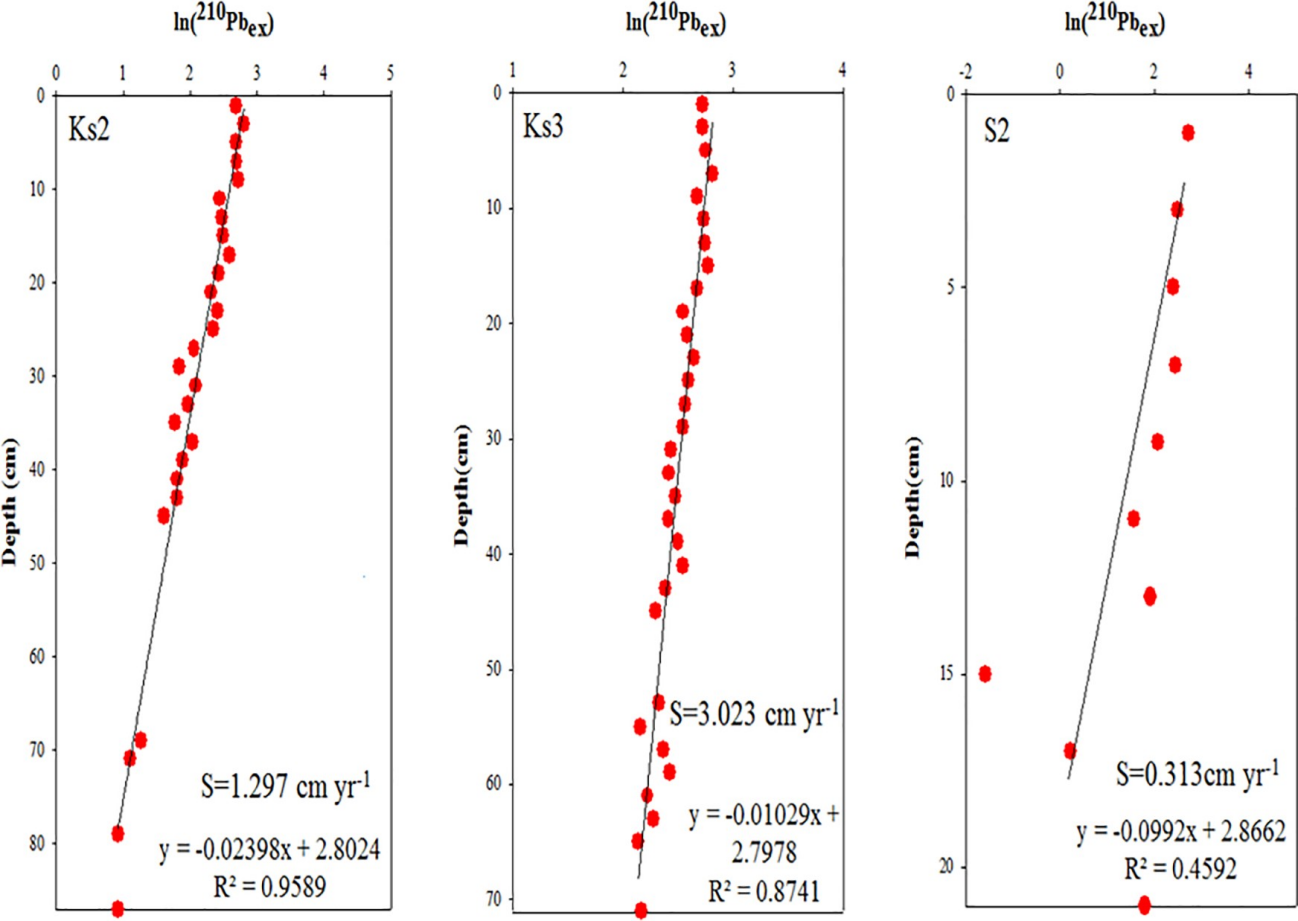
Sedimentation rates of three core sediments derived from the relationship between excess ^210^Pb and depth.

The distribution of clay and mud in Core Ks2, which was the closest to the major vents (approximately 254 m), ranged from 10% to 20% and from 95.8% to100%, respectively (Fig 2A). TS was relatively low at 26–60 cm, and its distribution ranged from 0.05 to 8.21 mg g^−1^ (Fig 2B). The distribution of TOC mirrored that of mud and ranged from 5.77 to 8.60 mg g^−1^ in Core Ks2 (Fig 2C). The vertical distributions of clay in Core Ks3 ranged from 11% to 26%, whereas that of mud ranged from 92% to approximately 100% (Fig 3A). The TS ranged from 2.89 to 8.64 mg g^−1^ (Fig 3B), and the TOC ranged from 4.80 to 9.11 mg g^−1^ in Core Ks3 (Fig 3C). Moreover, the variation in the TOC and TS of Core Ks3 was profound below 30 cm. The vertical distributions of clay and mud contents in Core S2 ranged from 18.7% to 31.2% for clay and from 76.7% to approximately 96.7% for mud (Fig 4A). The TS ranged from 0 to 4.62 mg g^−1^ (Fig 4B), and TOC ranged from 4.75 to 7.20 mg g^−1^ (Fig 4C). The variability of the fine particle, TOC, and TS contents was substantially larger in Core S2 than in Cores Ks2 and Ks3. Moreover, the sedimentation rate of Core Ks2 (1.297 cm yr^−1^) and Core Ks3 (3.023 cm yr^−1^) was rather high, whereas that of Core S2 (0.313 cm yr^−1^) was low, because Cores Ks2 and Ks3 were closer to the islet than Core S2 was (Fig 5). Nevertheless, the sedimentation was apparently steady in the three cores.

### Vertical distributions of metals in core sediments

**Fig 6:**
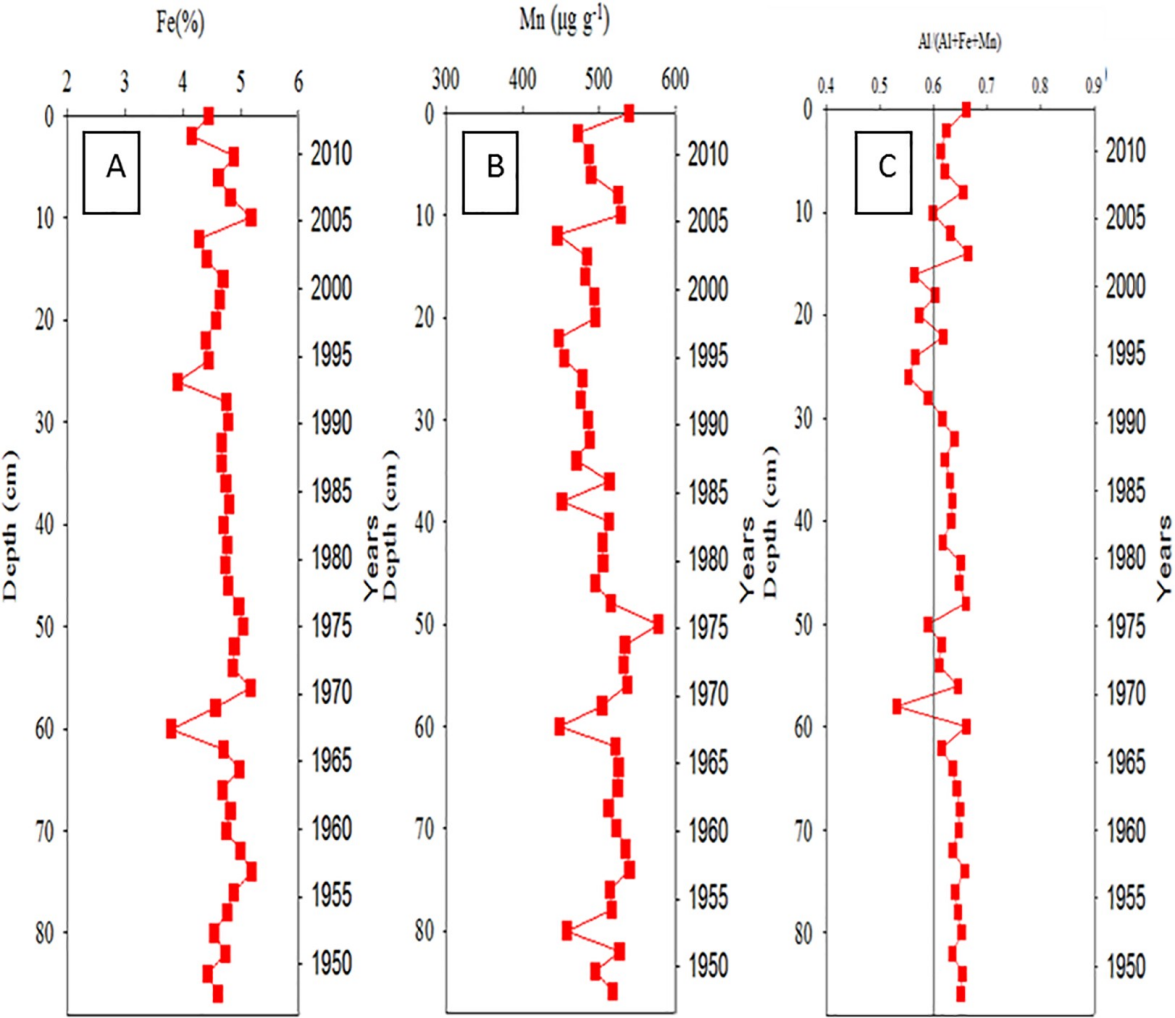
Temporal variations of Fe, Mn and the ratio of Al/(Al+Fe+Mn) in Core Ks2 sediments.

**Fig 7:**
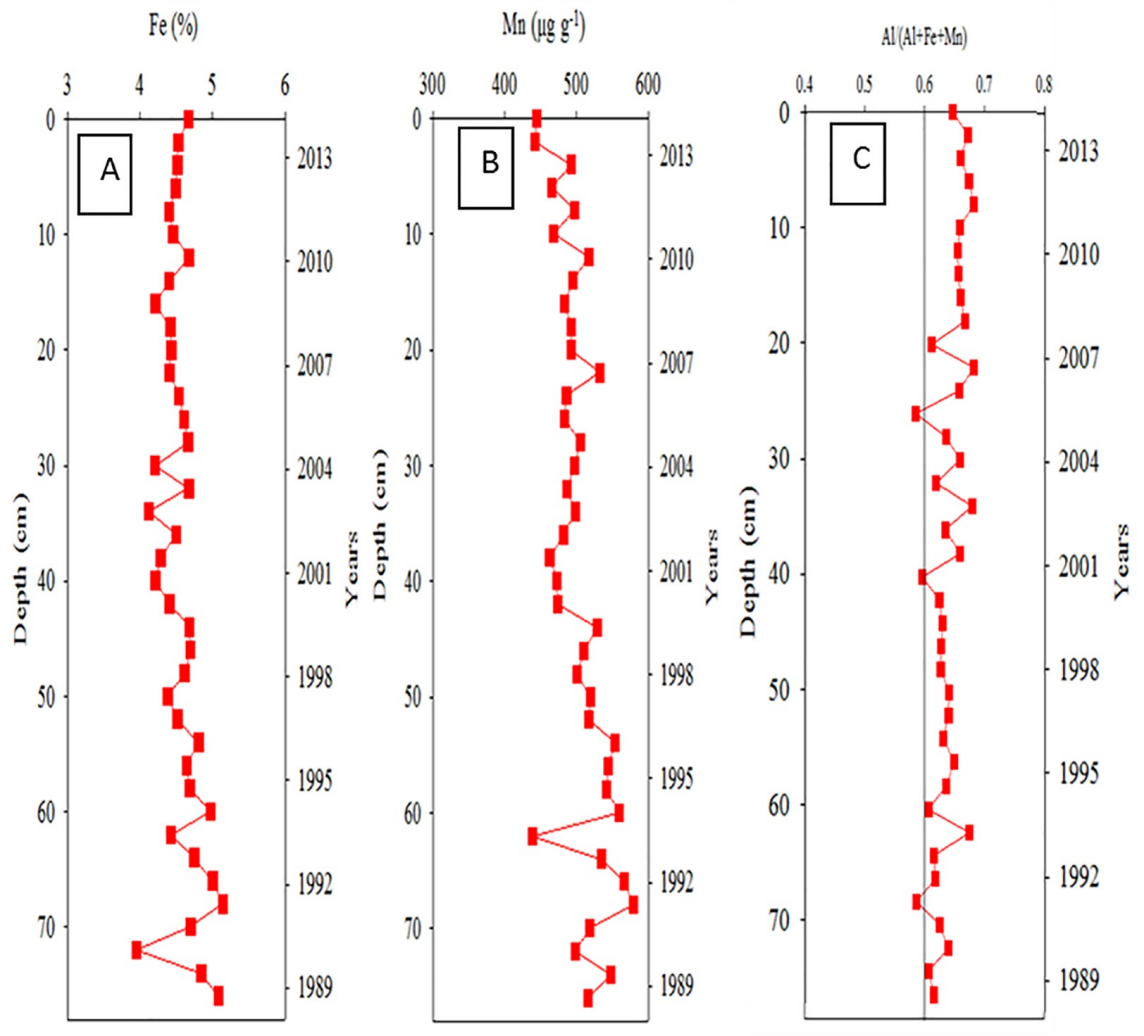
Temporal variations of Fe, Mn and the ratio of Al/(Al+Fe+Mn) in Core Ks3 sediments.

**Fig 8:**
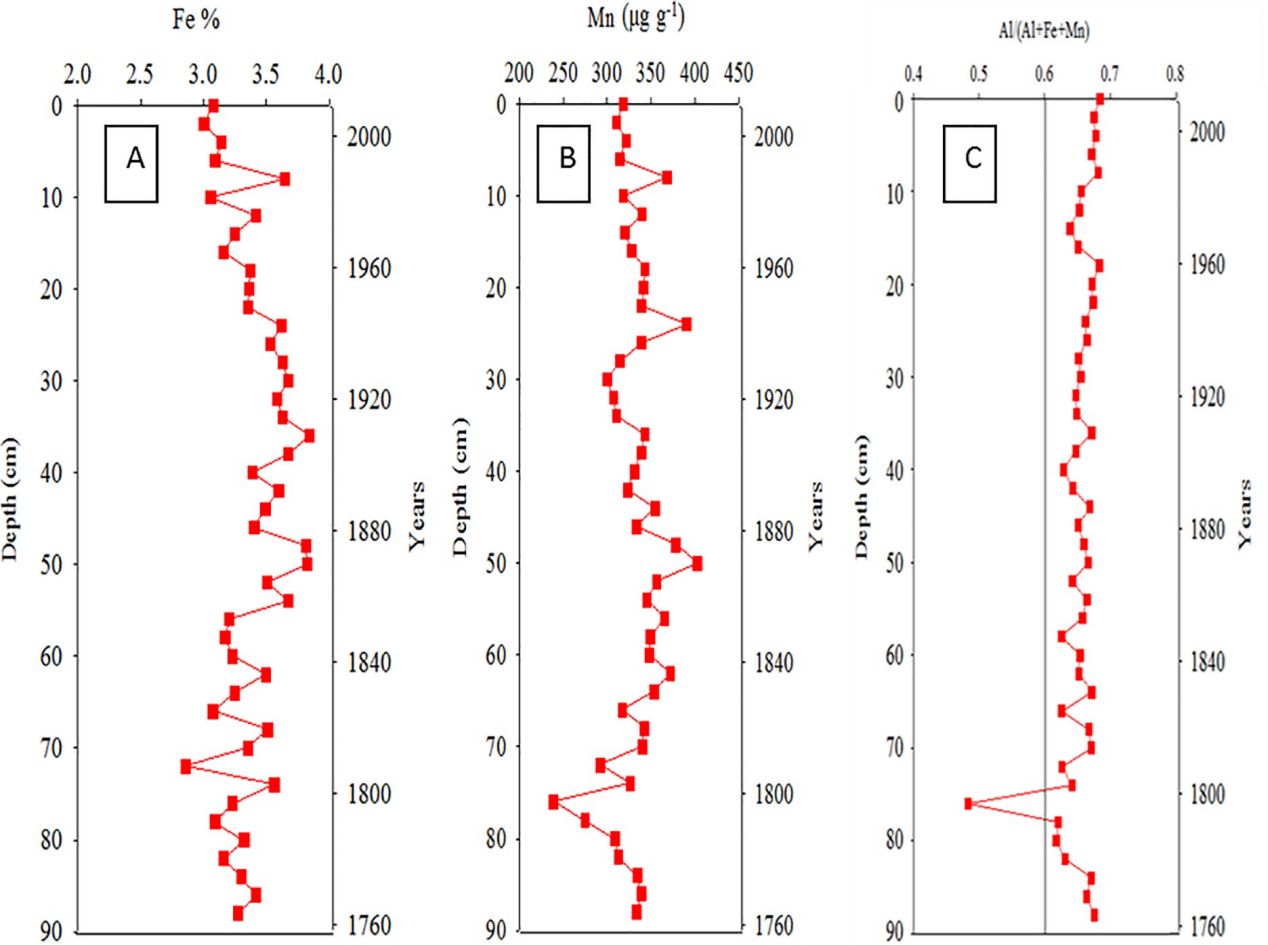
Temporal variations of Fe, Mn and the ratio of Al/(Al+Fe+Mn) in Core S2 sediments.

Fig 6A and 6B illustrate the vertical distributions of Fe and Mn (the hydrothermally derived metals) in Core Ks2. The total concentration of Fe and Mn ranged from 3.86% to 5.22% and from 454 to 540 μg g^−1^, respectively. Dramatic changes in these concentrations occurred at core depths of approximately 10, 25, and 60 cm. To enhance conciseness, the distributions of the other metals in Core Ks2 (Al, As, Ca, Mg, Mn, Co, Cu, Ni, Pb, and Zn) are shown in S1 Table. By contrast, the vertical change in Fe concentration was most evident below 60 cm and ranged from 3.95% to 5.08% in Core Ks3 (Fig 7A). Additionally, the concentration of Mn ranged from 442 to 579 μg g^−1^ and varied conspicuously below 42 cm (Fig 7B). The concentrations of the remaining metals are shown in S2 Table. In Core S2, the concentration of Fe and Mn ranged from 2.86% to 3.84% and from 238 to 402 μg g^−1^, respectively (Fig 8A and 8B). Moreover, neither Fe nor Mn demonstrated significant vertical changes within Core S2. The distributions of the other metals in S2 are presented in S3 Table. Overall, the vertical change in Al concentration in the three cores was relatively small because Al is a crust-derived metal and thus had very high initial concentrations [21, 25].

## Discussion

### Effects of physical and geochemical properties on concentrations of metals in sediments

**Fig 9:**
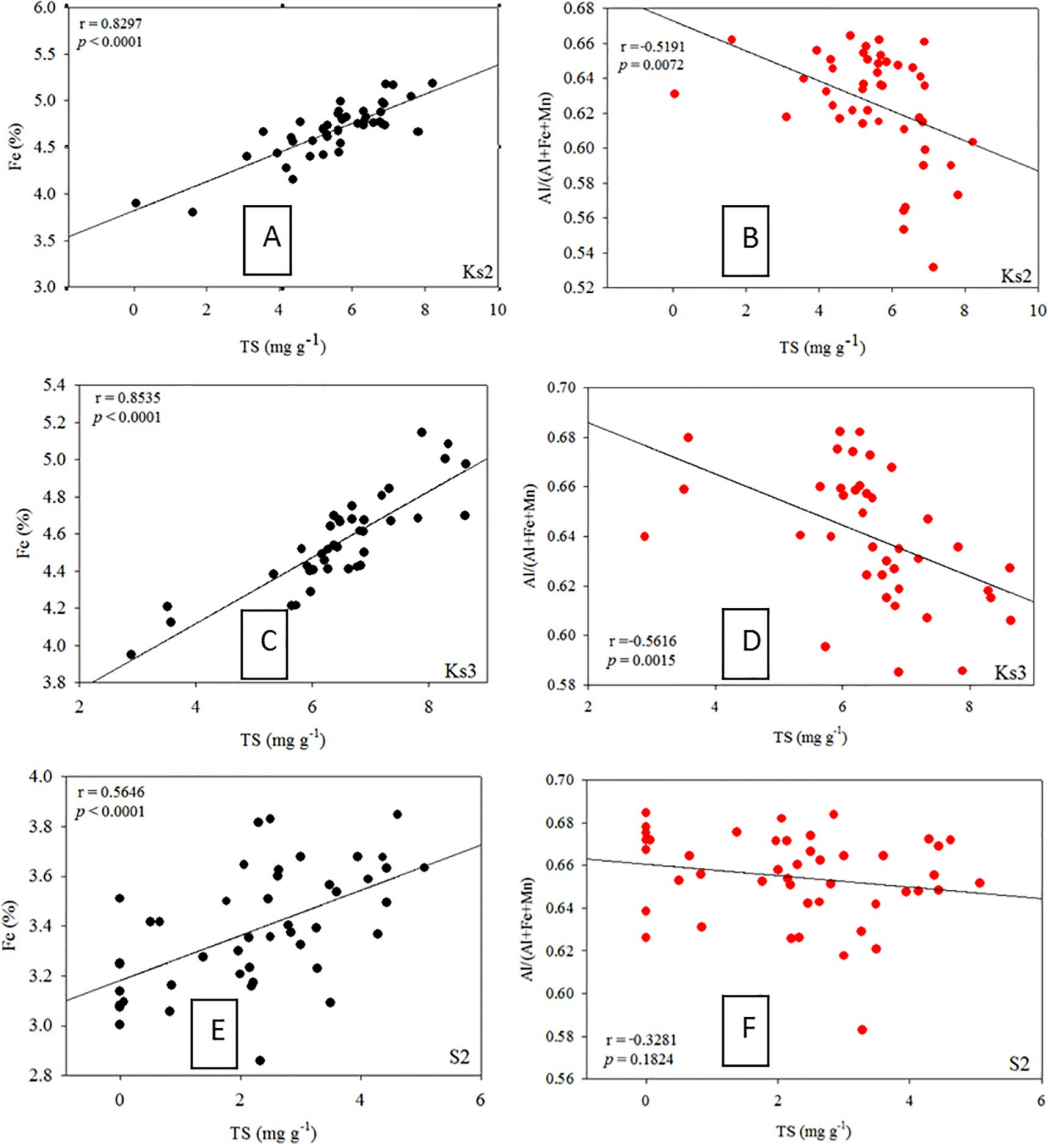
Correlations between TS and Fe and between TS and the ratio of Al/(Al+Fe+Mn) in three core sediments.

Particle-size and organic-matter content are generally regarded as two critical factors in controlling the distributions of metals in polluted and nonpolluted sediments [20−22]. Although hydrothermal discharge was the main source of the metals in the study area, as shown in Table 1, most metals did not deposit predominantly in the venting zone as hydrothermal plumes were influenced significantly by tidal current and Kuroshio Current [6, 10]. The tidal current moves generally from southeast to northwest during the spring tide and from north to south during the ebb tide, and the Kuroshio Current flows northward along the eastern coast of KST. Consequently, the influence of hydrothermalism on metal concentration cannot be derived simply from locations away from the venting source. Because fine particles and organic carbon are generally associated with an environment favorable for sedimentation, the accumulation of metals in sediments must be influenced by the strength of the source as well as by physical and geochemical properties. However, such influence cannot be determined merely from the total concentration of metals. Surprisingly, fine particles in Core Ks2 were nonsignificantly correlated with TOC, but were significantly correlated with TS (r > 0.345, p < 0.068), implying that hydrothermalism may have markedly affected the deposition of fine particles and organic carbon. Consequently, fine particles and TOC do not correlate significantly with metals in Core Ks2. In most coastal sediments, fine particles and organic carbon are significantly correlated with most metals in coastal seas off Taiwan [21, 22]. These results imply that Core Ks2 was affected by hydrothermalism; thus, the TS, which is an index of hydrothermalism was significantly correlated with Fe (r = 0.829, p < 0.0001; Fig 9A), Mn (r = 0.396, p < 0.05; figure omitted), Pb (r = 0.391, p < 0.05; figure omitted), Zn (r = 0.393, p < 0.05; figure omitted), Cu (r = 0.418, p < 0.01; figure omitted), Ni (r = 0.489, p < 0.01; figure omitted), and As (r = 0.527, p < 0.005; figure omitted). In addition, strong and significant positive correlations existed between Fe and As (r = 0.612, p < 0.0001) and between Mn and As (r = 0.669, p < 0.0001). Because the sedimentation analyzed was quite stationary (Fig 5), coastal erosion and slumping were excluded as causative factors. Therefore, the metals in Core Ks2 were more strongly affected by their hydrothermal source than by geochemical properties.

In Core Ks3, fine particles correlated significantly with TOC (r > 0.400, p < 0.008) and with TS (r > 0.494, p < 0.0007), which may imply that Core Ks3 was not affected by hydrothermalism as strong as Core Ks2 was. However, a significant correlation (r = 0.854, p < 0.0001) was revealed between Fe and TS (Fig 9C). Moreover, all other metals except Cu were correlated significantly with TS (r > 0.352, p < 0.05). Fe and Mn strongly correlated with As (r > 0.637, p < 0.0001), which was also significantly correlated with TS (r = 0.505, p = 0.0028). These results suggest that the vertical distribution of the metals in Core Ks3 was controlled by both geochemical processes and hydrothermalism.

Compared with Cores Ks2 and Ks3, the TS in Core S2 sediments decreased conspicuously; therefore, the effects of hydrothermalism on Core S2 may be smaller than those on Cores Ks2 and Ks3. This result is consistent with the location of the cores, because Core S2 was located far from the vents. Consequently, all metals except Mn were positively correlated with fine particles and TOC (r > 0.314, p < 0.05). In addition, a significant correlation was also revealed between Fe and TS in Core S2 (r = 0.565, p < 0.0001; Fig 9E). All metals were positively correlated with TS (r > 0.337, p < 0.05) except for a correlation between Mn and TS. Moreover, all metals correlated well with each other (r > 0.407, p < 0.02) except for Ni and Pb which correlated poorly with certain metals. The vertical distribution of Ca was much lower in Cores Ks2 (0.77%–1.43%), Ks3 (0.88%–1.71%), and S2 (0.55%–1.40%) than in the crust [25] and coastal sediments in Taiwan [21, 22]. The concentration of Mg (0.56%–1.46%) was also much lower than that in the crust (2.33%) [25]. Relatively low concentrations of Ca and Mg may be attributable to leaching carbonates caused by acidic hydrothermal plumes. Nevertheless, hydrothermalism likely affected Core S2 less than it did affect Cores Ks2 and Ks3.

#### Indications of hydrothermal impacts on core sediments

Previous studies revealed that the ratios of Al/(Al+Fe+Mn) and TS content were strong indicators of hydrothermalism on sediments [10, 29, 30]. Hydrothermal activity generally discharges high concentrations of dissolved Fe, Mn, and Al, but dissolved Fe is oxidized and precipitated more quickly than dissolved Mn and Al [14, 18]. Moreover, the concentration of Al in sediments may not be affected by hydrothermal discharge because the precipitation of dissolved Al is small compared to the original content in sediments that is quite high in marine sediments. As a result, the Al/(Al+Fe+Mn) ratio increases and the TS decreases with decreasing emission strength, or increasing distance between surface sediments and the venting source. Such relationship appears to be true for different venting systems. Boström and Peterson [29] found an increase of Al/(Al+Fe+Mn) ratio in sediments with distance away from the venting center on the East Pacific Rise. The Al/(Al+Fe+Mn) ratio may fall between 0.001 and 0.6 for sediments under the influence of hydrothermalism and the ratio was greater than 0.6 for sediments without the influence of hydrothermalism or for sediments derived mainly from river sources [31, 32]. In this study, the average Al/(Al+Fe+Mn) ratio was below 0.69 (Figs 2C, 3C and 4C), indicating that the sediments must have been influenced by a different strength of hydrothermalism. Additionally, a strong indicator of hydrothermal effects is that the Al/(Al+Fe+Mn) ratio was significantly inversely correlated with TS in Core Ks2 (r = −0.519, p = 0.007; Fig 9B) and Core Ks3 (r = −0.562, p = 0.0015; Fig 9D). However, it was poorly correlated with TS in Core S2 (r = −0.322, p = 0.182; Fig 9F), because Core S2 was least influenced by hydrothermalism.

#### Comparison of emission-index chronology in three cores

**Fig 10:**
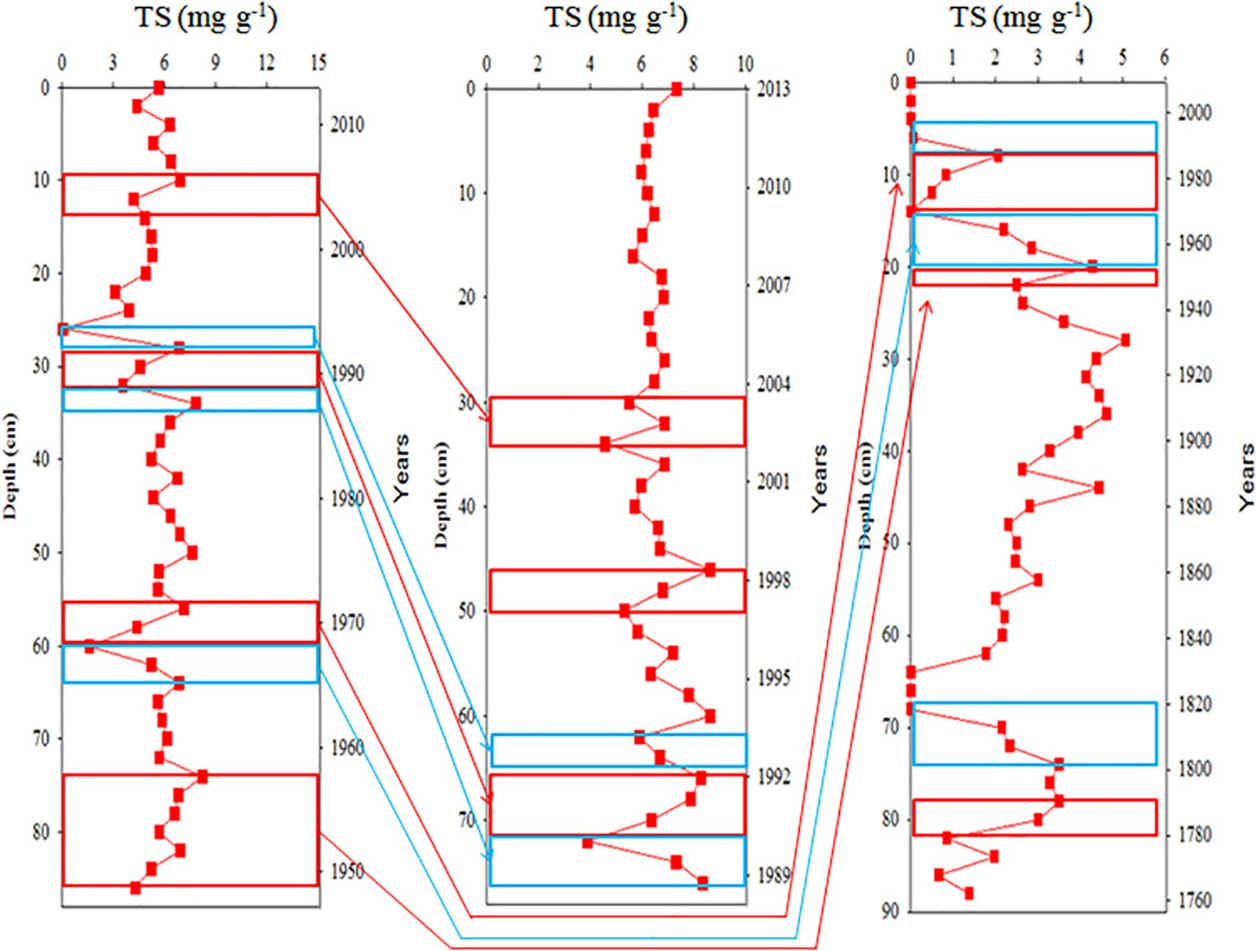
Temporal variations TS in three core sediments and similar time spans of increased or decreased trends of TS among three cores. From left to right panels are Core Ks2, Ks3 and S2, respectively.

The records of emission strength in the three cores were compared using TS as the index of emission strength (Fig 10). TS in Core Ks2, which is the core closest to the vents, increased during 1950–1956, 1968–1970, 1982–1987, 1990–1992, and 2004–2005, but decreased in 1967–1968, 1988–1990, and 1994–1995. The temporal changes in the TS of Cores Ks3 and S2 were fairly identical with those of Core Ks2 within the same time span.

Certain natural and anthropogenic forces must affect hydrothermalism and simultaneously determine the temporal variations of hydrothermalism in the three cores. Previous studies have indicated that tidal pumping may considerably affect semidiurnal variations in submarine hydrothermal discharge of fluids [6, 33, 34]. However, no long-term observation data were available for determining the interannual changes in KST hydrothermal discharge. For a time span much shorter than the period since the most recent eruption (approximately 7 ka BP), the episodic pulses of hydrothermal discharge derived from temporal variations of geochemical signals may be primarily caused by natural forces, because no human perturbation such as mining occurred there. Commercial mining was generally regarded as the major anthropogenic activity to have impacts on the venting system [35, 36]. Other than that, scientific research may be also a source of anthropogenic disturbance but it is negligible in the KST hydrothermal system because the current research activity is not allowed to alter the vent environment. Thus, we propose that local typhoons and large earthquakes are natural forces that affect hydrothermal discharge indirectly.

To determine cause and effect, we identified large earthquakes (M_L_ > 5) with epicenters that were located in northeastern Taiwan. Such earthquakes occurred in 1910 (April), 1920 (June), 1922 (September), 1951 (October–November), 1966 (March), 1967 (October), 1982 (January), 1990 (December), 2004 (May), 2009 (December), 2015 (April), and 2016 (February and May) [37]. The occurrence of big earthquakes was fairly closely related to the time spans in which hydrothermal discharge and sulfide content increased, despite the possible time lag between the start of the increase and the date when the earthquake occurred. A gradual increase was observed in borehole temperatures between 180 and 270 m in borehole depth after an earthquake occurred on September 14, 2006 [38]. The researchers believed that fluid flow along the Neutou fault in northeastern Taiwan was influenced by the earthquake. Johnson et al. [39] reported an increase in temperature and fluid output following a modest earthquake on the Juan de Fuca mid-ocean ridge, although the relationship remains highly arguable and misunderstood. In hydrothermal systems, seismic activity may open fractures, depending on the strength of the earthquake, and hot water in the vicinity of magma could flow upwards to the surface through these fractures [40]. A recent study also suggested that earthquakes can trigger the eruption of mud in Taiwan, Japan and other countries [41]. Nozaki et al. [42] reported the rapid growth of sulfide chimneys associated with seismic events in some hydrothermal fields. Gamo et al. [43] deployed an *in situ* automatic chemical analyzer in the Sagami Bay (Japan) and detected an enhanced Mn flux up to 10 times that of the background level followed by a M5.8 earthquake. Nakayama et al. [44] also found considerable enrichments of Mn and Fe in the bottom waters (>10 times higher than the background level) of Japan Trench subduction zone after a M7.5 earthquake. Submarine crustal deformations induced by earthquakes may play a critical role in supplying elevated chemical fluxes from the seafloor through local faults and fissures.

On the other hand, typhoons striking KST may cause land slides and debris to flow from the islet or due to coastal erosion, which may result in the closing or clogging of the hydrothermal vents. Two intermediate typhoons occurred in 1967, three intermediate typhoons occurred in 1990, and one strong typhoon occurred in 1994 [45], consistent with the periods during which venting activity diminished and sulfide content in sediments decreased. Although no historical data are available to support this scenario, local divers did experience the disappearance of certain vents after typhoons passing through the study area [46]. Typhoons also brought in huge rain and caused resuspension of shallow bottom sediments and turbidity flow from Taiwan’s rivers [47]. Because KST Islet is just 10 km away from the Lanyang River (river mouth: 121.836°E, 24.704°N) which is the largest river in eastern Taiwan, it is reasonable to project a considerable amount of terrestrial sediments brought in the venting field directly by typhoons or through erosion of previous deposits. This hypothesis, however, should be tested and approved further through a long-term observation, particularly after typhoons and earthquakes.

### Conclusion

The vertical distributions and accumulation of sulfide and metals in KST-associated sediments were evidently influenced by submarine hydrothermal discharge, because venting fluids supplied considerable amounts of dissolved sulfide and metals without human perturbation. Geochemical indices of hydrothermalism, namely TS and the Al/(Al+Fe+Mn) ratio, were effective in elucidating a centennial scale of change in submarine hydrothermalism. Correlations were significantly positive between TS and Fe, and significantly negative between TS and the Al / (Al + Fe + Mn) ratio in the three cores, at different distances from the vents, showing the influence of hydrothermalism on venting field sediments. The TS concentration as well as the hydrothermal discharge in Core Ks2 increased during the five time spans, but decreased during three time spans for a total time span of 70 years. The temporal variations of TS concentration in Core Ks3 and S2 were very similar with that of Core Ks2, supporting strongly the evidence of temporal changes in hydrothermalism. The emission strength was apparently reflected in the temporal variation of emission indices. These temporal variations of hydrothermal discharge are likely determined by the impacts of typhoons and large earthquakes.

## Supporting information

S1 Table Concentrations of various metals in Core Ks2 Sediments. (DOC)

S2 Table Concentrations of various metals in Core Ks3 Sediments. (DOCX)

S3 Table Concentrations of various metals in Core S2 Sediments. (DOCX)

S1 Fig Comparison of sulfide spreading zone between 2012 and 2016 on the surface water off the eastern head of Kueishantao Islet. (DOCX)

## References

[1] Tarasov VG, Gebruk AV, Mironov AN, Moskalev LI. Deep-sea and shallow-water hydrothermal vent communities: Two different phenomena? Chem Geol. 2005; 224:5–39.

[2] Chang NN, Lin LH, Tu TH, Jeng MS, Chikaraishi Y, Wang PL. Trophic structure and energy flow in a shallow-water hydrothermal vent: Insights from a stable isotope approach. Plos One 2018; 10.1371/journal.pone.0204753 30332427 10.1371/journal.pone.0204753 PMC6192584

[3] Chen YG, Wu WS, Chen CH, Liu TK. A date for volcanic eruption inferred from a siltstone xenoliths. Quat Sci Rev. 2001; 20:869–873.

[4] Chiu CL, Song SR, Hsieh YC, Chen CX. Volcanic characteristics of Kueishantao in northeast Taiwan and their implications. Terr Atmos and Ocean Sci. 2010; 21:575–585.

[5] Yang TF, Lan TF, Lee HF, Fu CC, Chuang PC, Lo CH, et al Gas compositions and helium isotopic ratios of fluid samples around Kueishantao, NE offshore Taiwan and its tectonic implications. Geochem J. 2005; 39:469–480.

[6] Chen CTA, Zeng ZG, Kuo FW, Yang BJ, Tu YY. Tide-influenced acidic hydrothermal system offshore NE Taiwan. Chem Geol. 2005; 224:69–81.

[7] Chen YJ. Wu JY, Chen CTA, Liu LL. Effects of low-pH stress on shell traite of the dove snail, *Anachis misera*, inhabiting shallow-vent environments off Kueishan Islet, Taiwan. Biogeosciences 2015; 12: 2631–2639.

[8] Chen XG, Lyu SS, Garbe-Schӧnberg D, Lebrato M, Li X, Zhang HY, et al Heavy metals from Kueishantao shallow-sea hydrothermal vents, offshore northeast Taiwan. J Mar Syst. 2016; 10.1016/j.marsys.2016.11.018

[9] Han C., Ye Y., Pan Y., Qin H., Wu G., Chen C. T. A. Spatial distribution pattern of seafloor hydrothermal vents to the southeastern Kueishan Tao offshore Taiwan Island. Acta Oceanol Sin. 2014 33:37–44.

[10] Hung JJ, Yeh HY, Peng SH, Chen CTA. Influence of submarine hydrothermalism on sulfur and metal accumulation in surface sediments in the Kueishantao venting field off northeastern Taiwan. Mar Chem. 2018; 198:88–96.

[11] McCarthy KT, Pichler T, Price R. Geochemistry of Champagne Hot Springs shallow hydrothermal vent field and associated sediments, Dominica, Lesser Antilles. Chem Geol. 2005; 224:55–68.

[12] Price R, Savov I, Friedrich BP, Buhring SI, Amend J, Pichler T. Processes influencing extreme As enrichment in shallow-sea hydrothermal fluids of Milos Island, Greece. Chem Geol. 2013; 348:15–26.

[13] Price RE, Pichler T. Distribution, speciation and bioavailability of arsenic in a shallow-water submarine hydrothermal system, Tutum Bay, Ambitle Island, PNG. Chem Geol. 2005; 224:122–135.

[14] Pichler T, Veizer J, Hall G. The chemical composition of shallow-water hydrothermal fluid in Tutum Bay, Ambite Island, and their effect on ambient seawater. Mar Chem. 1999; 64, 229–252.

[15] Cardigos F, Colaco A, Dando PR, Avila SP, Sarradin PM, Tempera F, et al Shallow water hydrothermal vent field fluids and communities of the D. João de Castro Seamount (Azores). Chem. Geol. 2005; 224, 153–168.

[16] Sedwick P, Stüben D. Chemistry of shallow submarine warm springs in an arc-volcanic setting: Vulcano Island, Aeolian Archipelago, Italy. Mar Chem. 1996; 53:146–161.

[17] Sander S, Koschinsky A. Metal flux from hydrothermal vents increased by organic complexation. Nat Geosci. 2011; 4:145–150.

[18] Bruland KW, Middag R, Lohan MC. Controls of Trace Metals in Seawater In: Holland H., Turekian K. (Eds.), vol. 8, 2014; Treatise on Geochemistry. Elsevier, Amsterdam

[19] Lavelle JW, Cowen JP, Massoth GJ. A Model for the Deposition of Hydrothermal Manganese Near Ridge Crests. J Geophy Res. 1992; 97(C5):7413–7427.

[20] Mason RP. Trace metals in aquatic systems Wiley-Blackwell, pp 219–309. Chichester, UK; 2013.

[21] Hung JJ, Hsu CL. Present state and historical changes of trace metal pollution in Kaoping coastal sediments, southwestern Taiwan. Mar Pollut Bull. 2004; 49:986–998. 10.1016/j.marpolbul.2004.06.028 15556185 10.1016/j.marpolbul.2004.06.028

[22] Hung JJ, Lu CT, Huh CA, Liu JT. Geochemical control on distributions and speciation of As and Hg in sediments along the Kaoping Estuary-Canyon system off southwestern Taiwan. J Mar Syst. 2009; 76:479–495.

[23] Fonselius SH. Determination of hydrogen sulfide In: Grasshoff K. (Ed.), Methods of Seawater Analysis. Verlag Chemie 1983; pp. 73–80.

[24] Ho TY, Chien CT, Wang BN, Siriraks A. Determination of trace metals in seawater by an automated flow injection ion chromatograph pretreatment system with ICPMS. Talanta 2010; 82:1478–1484 10.1016/j.talanta.2010.07.022 20801359 10.1016/j.talanta.2010.07.022

[25] Chan I, Hung JJ, Peng SH, Tseng LC, Ho TY, Hwang JS. Comparison of metal accumulation in the azooxanthellate scleractinian coral (*Tubastraea coccinea*) from different polluted environments. Mar Pollut Bull. 2014; 85:648–658. 10.1016/j.marpolbul.2013.11.015 24321880 10.1016/j.marpolbul.2013.11.015

[26] Huh CA, Su CC, Wang CH, Lee SY, Lin IT. Sedimentation in the southern Okinawa Trough—rates, turbidites and a sediment budget. Mar Geol. 2006; 231:129–139.

[27] Varnavas S.P., Cronan D.S. Submarine hydrothermal activity off Santorini and Milos in the central Hellenic volcanic arc: a synthesis. Chem Geol. 2005; 224: 40–54.

[28] Von Dann KL, Edmond JM, Grant B, Measures CI. Chemistry of submarine hydrothermal solution at 21 N, East Pacific Rise. Geochim. Cosmochim. Acta 2005; 49: 2197–2220.

[29] Boström K, Peterson MNA. Precipitates from hydrothermal exhalations on the East Pacific Rise. Econ Geol. 1966; 61:1258–1265.

[30] Mascarenhas Peereira MBL, Nath BN. Selective leaching studies of sediments from a seamount frank in the Central Indian basin: Resolving hydrothermal, volcanogenic and terrigenous components using major, trace and rare-earth elements. Mar Chem. 2010; 121: 49–66.

[31] Yamamoto K. Geochemical characteristics and depositional environments of cherts and associated rocks in the Franciscan and Shimanto Terrances. Sed Geol. 1987; 52:65–108.

[32] Du YS, Zhu J, Gu SZ, Xu YJ, Yang JH. Sedimentary geochemistry of the Cambrian-Ordovician cherts: Implication on archipelagic ocean of North Qilian orogenic belt. Sci. in China Ser. D: Earth Sci. 2007; 50:1628–1644.

[33] Little SA, Stolzenbach KD, Grassle FJ. Tidal current effects on temperature in diffuse hydrothermal flow: Guaymas Basin. Geophy Res Lett. 1998; 15:1491–1494.

[34] Glasby GP, Kasahara J. Influence of tidal effects on the periodicity of earthquake activity in diverse geological settings with particular emphasis on submarine hydrothermal systems. Earth-Sci Rev. 2001; 52:262–297.

[35] Van Dover CL. Impacts of anthropogenic disturbances at deep-sea hydrothermal vent ecosystems: A review. Mar Environ Res. 2014; 102:59–72. 10.1016/j.marenvres.2014.03.008 24725508 10.1016/j.marenvres.2014.03.008

[36] Van Dover CL, Ardron JA, Escobar E, Gianni M, Gjerde KM, Jaeckel A, et al Biodiversity loss from deep-sea mining. Nature Geosci. 2017; 10:464–465.

[37] Central Weather Bureau, Taiwan. http://www.cwb.gov.tw/V7e/earthquake/, 2016a.

[38] Chiang HT, Shyu CT, Chang HI, Tsao S, Chen CX. Geothermal Monitoring of Kueishantao Island Offshore of Northeastern Taiwan. Terr. Atmos. Ocean. Sci. 2010; 21: 563–573.

[39] Johnson HP, Hutnak M, Dziak RP, Fox CG, Urcuyo I, Cowen JP, et al Earthquake-induced changes in a hydrothermal system on the Juan de Fuca mid-ocean ridge. Nature 2000; 407:174–177. 10.1038/35025040 11001052 10.1038/35025040

[40] Silver PG, Valette-Silver JN. A spreading episode at the southern end of the San Andreas fault system. Nature 1987; 326:589–593.

[41] Bonini M, Rudolph ML, Manga M. Long- and short-term triggering and modulation of mud volcano eruptions by earthquakes. Tectonophysics 2016; 672−673: 190–211.

[42] Nozaki T, Ishibashi J-I, Shimada K, Nagase T, Takaya Y, Kato Y, et al Rapid growth of mineral deposits at artificial seafloor hydrothermal vents. Sci. Reports 2016; 10.1038/srep22163 26911272 10.1038/srep22163 PMC4766430

[43] Gamo T, Okamura K, Mitsuzawa K, Askawa K. Tectonic pumping: earthquake-induced chemical flux detected in situ by a submarine cable experiment in Sagami bay, Japan. Proc Jpn Acad Ser B Ohys Biol Sci 2007; 83: 199–204. 10.2183/pjab/83.199 PMC3859272 24367146

[44] Nakayama E, Maruo M, Obata H, Isshiki K, Okamura K, Gamo T, et al In: Marine Environment: the past, present and future (ed. Chen C.-T. A.). The Fuwen Press, Kaohsiung (Taiwan) 2002; pp. 345–355

[45] Central Weather Bureau, Taiwan. http://rdc28.cwb.gov.tw/tylist_warning.php, 2016b.

[46] Dahma HU, Schizas NV, James RA, Wang L, Hwang JS. Marine hydrothermal vents as templates for global change scenarios. Hydrobiologia 2018; 10.1007/s10750-017-3295-z

[47] Liu JT, Wang YH, Yang RJ, Hsu RT, Kao SJ, Lin HL, et al Cyclone-induced hyperpycnal turbidity currents in a submarine canyon. J Geophy Res 2012; 10.1029/2011JC007630

