# Supplementary material for: External-forcing modulation on temporal variations of hydrothermalism-evidence from sediment cores in a submarine venting field off northeastern Taiwan
Source: PLoS One. 2018 Nov 29;13(11):e0207774. doi: 10.1371/journal.pone.0207774 (PMC6264505; doi:10.1371/journal.pone.0207774)
Supplement: S1 Table — (DOC) [file pone.0207774.s001.doc]

**S1 Table.** Concentrations of various metals in Core Ks2 Sediments

| Depth | Al | As | Ca | Mg | Mn | Co | Cu | Ni | Pb | Zn |
| --- | --- | --- | --- | --- | --- | --- | --- | --- | --- | --- |
| (cm) | % | μg g^-1^ | % | % | μg g^-1^ | μg g^-1^ | μg g^-1^ | μg g^-1^ | μg g^-1^ | μg g^-1^ |
| 0~2 | 8.81 | 12.63 | 1.29 | 1.36 | 539.5 | 11.64 | 24.60 | 33.60 | 23.25 | 74.55 |
| 2~4 | 6.98 | 11.85 | 1.21 | 1.08 | 478.3 | 11.04 | 23.10 | 37.50 | 20.85 | 72.45 |
| 4~6 | 7.85 | 13.92 | 1.36 | 1.33 | 524.6 | 11.52 | 25.20 | 37.20 | 22.65 | 77.55 |
| 6~8 | 7.64 | 14.21 | 0.88 | 1.28 | 458.4 | 11.46 | 28.50 | 35.25 | 22.65 | 77.85 |
| 8~10 | 9.28 | 15.90 | 1.05 | 1.34 | 486.9 | 12.29 | 26.85 | 35.40 | 24.15 | 79.50 |
| 10~12 | 7.80 | 16.05 | 1.25 | 1.12 | 513.6 | 11.90 | 27.45 | 37.35 | 22.95 | 77.85 |
| 12~14 | 7.43 | 13.46 | 1.31 | 1.18 | 504.0 | 10.50 | 21.90 | 31.80 | 18.00 | 68.55 |
| 14~16 | 8.81 | 12.60 | 1.34 | 1.30 | 484.6 | 10.35 | 20.85 | 30.90 | 18.00 | 67.65 |
| 16~18 | 6.14 | 12.80 | 1.07 | 1.02 | 482.3 | 10.67 | 21.60 | 31.35 | 19.50 | 71.70 |
| 18~20 | 7.12 | 13.73 | 1.14 | 1.21 | 448.5 | 10.61 | 21.90 | 32.10 | 20.25 | 74.10 |
| 20~22 | 6.19 | 13.95 | 0.90 | 1.00 | 454.6 | 10.58 | 20.85 | 33.00 | 18.60 | 69.00 |
| 22~24 | 7.19 | 10.92 | 1.39 | 1.24 | 489.6 | 10.44 | 19.80 | 30.15 | 18.15 | 69.30 |
| 24~26 | 5.85 | 11.10 | 1.29 | 0.64 | 515.4 | 10.82 | 21.15 | 32.70 | 19.65 | 71.70 |
| 26~28 | 4.89 | 12.20 | 0.90 | 0.56 | 445.5 | 10.38 | 20.57 | 31.70 | 20.08 | 66.81 |
| 28~30 | 6.89 | 11.72 | 1.02 | 1.14 | 476.0 | 10.28 | 22.95 | 29.55 | 19.05 | 65.55 |
| 30~32 | 7.77 | 13.31 | 1.26 | 1.34 | 514.7 | 11.48 | 25.05 | 37.35 | 21.15 | 77.25 |
| 32~34 | 8.37 | 13.05 | 1.06 | 1.27 | 488.1 | 11.40 | 23.25 | 35.10 | 21.00 | 74.25 |
| 34~36 | 7.74 | 13.34 | 1.05 | 1.28 | 470.5 | 11.52 | 23.40 | 34.65 | 21.15 | 75.30 |
| 36~38 | 8.19 | 15.00 | 0.89 | 1.28 | 505.0 | 12.36 | 24.75 | 37.20 | 21.90 | 77.55 |
| 38~40 | 8.46 | 14.45 | 1.18 | 1.36 | 494.9 | 11.15 | 23.55 | 33.90 | 20.85 | 73.65 |
| 40~42 | 8.21 | 15.75 | 1.21 | 1.23 | 521.1 | 11.91 | 24.90 | 35.70 | 22.05 | 76.05 |
| 42~44 | 7.77 | 14.25 | 1.22 | 1.26 | 524.8 | 11.25 | 24.30 | 34.35 | 21.15 | 73.80 |
| 44~46 | 8.92 | 14.64 | 1.37 | 1.41 | 528.9 | 11.04 | 23.70 | 37.65 | 20.10 | 70.95 |
| 46~48 | 8.91 | 14.67 | 1.09 | 1.41 | 495.3 | 11.03 | 23.85 | 34.20 | 20.40 | 71.70 |
| 48~50 | 9.78 | 15.45 | 1.01 | 1.44 | 534.1 | 12.87 | 27.60 | 39.90 | 22.50 | 82.95 |
| 50~52 | 7.33 | 27.75 | 0.95 | 1.05 | 493.7 | 19.35 | 41.40 | 62.10 | 36.45 | 129.30 |
| 52~54 | 7.88 | 19.35 | 0.85 | 1.39 | 472.7 | 15.75 | 33.00 | 34.35 | 38.10 | 106.50 |
| 54~56 | 7.69 | 17.40 | 0.77 | 1.11 | 451.4 | 15.30 | 37.95 | 34.80 | 34.95 | 96.90 |
| 56~58 | 9.50 | 19.35 | 0.83 | 1.34 | 447.5 | 18.15 | 38.55 | 39.90 | 40.35 | 114.30 |
| 58~60 | 5.24 | 13.61 | 1.20 | 0.65 | 512.9 | 14.78 | 30.90 | 31.20 | 30.60 | 85.80 |
| 60~62 | 7.53 | 11.88 | 1.08 | 1.27 | 485.7 | 14.42 | 31.80 | 31.05 | 30.00 | 98.85 |
| 62~64 | 7.58 | 12.77 | 1.14 | 1.19 | 505.2 | 15.30 | 33.60 | 32.40 | 32.10 | 94.65 |
| 64~66 | 8.82 | 13.59 | 1.29 | 1.46 | 540.1 | 16.35 | 33.00 | 34.35 | 33.45 | 105.90 |
| 66~68 | 8.53 | 12.38 | 1.21 | 1.31 | 532.2 | 15.90 | 42.15 | 36.75 | 35.70 | 149.70 |
| 68~70 | 9.02 | 11.61 | 1.17 | 1.33 | 512.4 | 16.65 | 40.20 | 35.40 | 41.55 | 117.60 |
| 70~72 | 8.81 | 13.91 | 1.10 | 1.34 | 522.8 | 16.65 | 34.95 | 34.65 | 34.80 | 108.30 |
| 72~74 | 8.82 | 14.19 | 0.97 | 1.28 | 537.0 | 16.80 | 32.85 | 34.95 | 34.65 | 102.45 |
| 74~76 | 10.08 | 16.50 | 0.84 | 1.28 | 578.1 | 18.60 | 37.20 | 39.45 | 38.70 | 149.10 |
| 76~78 | 8.79 | 13.82 | 1.14 | 1.32 | 534.3 | 16.65 | 34.65 | 33.90 | 32.85 | 104.25 |
| 78~80 | 8.78 | 13.88 | 1.09 | 1.20 | 516.4 | 16.20 | 33.90 | 32.70 | 36.45 | 103.80 |
| 80~82 | 8.64 | 10.23 | 0.96 | 1.15 | 494.8 | 15.90 | 38.55 | 33.30 | 22.65 | 103.05 |
| 82~84 | 8.35 | 13.64 | 1.34 | 1.33 | 526.9 | 15.45 | 34.35 | 29.85 | 33.30 | 96.00 |
| 84~86 | 8.47 | 13.92 | 1.53 | 1.36 | 525.5 | 15.60 | 33.75 | 30.90 | 33.90 | 102.90 |
| 86~88 | 8.67 | 13.62 | 1.34 | 1.33 | 517.4 | 16.05 | 34.95 | 36.90 | 36.60 | 101.70 |
