# Supplementary material for: External-forcing modulation on temporal variations of hydrothermalism-evidence from sediment cores in a submarine venting field off northeastern Taiwan
Source: PLoS One. 2018 Nov 29;13(11):e0207774. doi: 10.1371/journal.pone.0207774 (PMC6264505; doi:10.1371/journal.pone.0207774)
Supplement: S2 Table — (DOCX) [file pone.0207774.s002.docx]

**S2 Table. Concentrations of various metals in Core Ks3 Sediments**

| Depth | Al | As | Ca | Mg | Mn | Co | Cu | Ni | Pb | Zn |
| --- | --- | --- | --- | --- | --- | --- | --- | --- | --- | --- |
| (cm) | % | μg g^-1^ | % | % | μg g^-1^ | μg g^-1^ | μg g^-1^ | μg g^-1^ | μg g^-1^ | μg g^-1^ |
| 0~2 | 8.64 | 7.59 | 1.59 | 1.32 | 444.7 | 13.54 | 55.97 | 25.97 | 27.27 | 64.20 |
| 2~4 | 9.39 | 6.80 | 1.73 | 1.47 | 442.5 | 13.23 | 50.72 | 25.82 | 26.52 | 64.35 |
| 4~6 | 8.87 | 12.26 | 1.44 | 1.37 | 492.9 | 13.21 | 28.82 | 32.57 | 29.07 | 71.10 |
| 6~8 | 9.38 | 12.80 | 1.57 | 1.41 | 465.4 | 13.56 | 53.12 | 33.77 | 28.17 | 66.60 |
| 8~10 | 9.55 | 13.19 | 1.48 | 1.42 | 497.1 | 13.45 | 58.52 | 34.82 | 28.92 | 74.55 |
| 10~12 | 8.68 | 11.45 | 1.48 | 1.33 | 467.7 | 13.15 | 52.07 | 41.72 | 29.82 | 58.50 |
| 12~14 | 8.99 | 16.95 | 1.08 | 1.13 | 517.5 | 14.44 | 65.12 | 37.52 | 39.12 | 75.60 |
| 14~16 | 8.51 | 13.20 | 1.84 | 1.40 | 495.4 | 13.53 | 57.77 | 33.02 | 29.67 | 67.80 |
| 16~18 | 8.27 | 11.99 | 1.42 | 1.30 | 483.0 | 12.40 | 53.57 | 28.52 | 25.77 | 60.15 |
| 18~20 | 8.98 | 13.53 | 1.53 | 1.45 | 492.4 | 13.36 | 53.42 | 31.52 | 28.17 | 68.85 |
| 20~22 | 7.06 | 13.20 | 1.07 | 1.00 | 492.7 | 12.82 | 53.42 | 31.97 | 30.57 | 93.90 |
| 22~24 | 9.57 | 15.15 | 1.02 | 1.45 | 532.4 | 13.30 | 53.27 | 36.02 | 32.22 | 77.10 |
| 24~26 | 8.79 | 13.53 | 1.25 | 1.33 | 486.1 | 12.84 | 51.02 | 36.02 | 26.97 | 64.35 |
| 26~28 | 6.58 | 11.70 | 1.28 | 0.94 | 483.5 | 12.51 | 48.92 | 28.97 | 27.42 | 60.30 |
| 28~30 | 8.22 | 14.36 | 1.11 | 1.13 | 505.3 | 14.37 | 52.37 | 33.62 | 31.02 | 71.10 |
| 30~32 | 8.22 | 12.20 | 1.40 | 1.30 | 497.8 | 11.92 | 46.82 | 29.57 | 24.72 | 58.50 |
| 32~34 | 7.66 | 19.80 | 1.30 | 1.23 | 486.7 | 13.27 | 53.12 | 49.82 | 31.92 | 64.65 |
| 34~36 | 8.85 | 12.14 | 1.57 | 1.38 | 498.7 | 12.46 | 56.12 | 31.82 | 27.12 | 99.90 |
| 36~38 | 7.90 | 13.08 | 1.25 | 1.30 | 481.9 | 13.27 | 56.27 | 33.32 | 30.72 | 71.40 |
| 38~40 | 8.38 | 12.63 | 1.22 | 1.36 | 463.0 | 12.12 | 51.47 | 28.52 | 29.22 | 61.50 |
| 40~42 | 6.27 | 11.34 | 1.28 | 0.86 | 472.6 | 10.99 | 28.37 | 32.27 | 25.32 | 68.70 |
| 42~44 | 7.41 | 13.31 | 0.95 | 1.28 | 474.3 | 12.97 | 29.42 | 31.37 | 30.12 | 67.95 |
| 44~46 | 8.06 | 14.87 | 0.97 | 1.27 | 528.6 | 12.16 | 25.52 | 26.42 | 25.62 | 77.85 |
| 46~48 | 7.98 | 18.30 | 1.11 | 1.22 | 510.7 | 14.59 | 34.07 | 60.62 | 31.62 | 114.60 |
| 48~50 | 7.83 | 17.70 | 1.05 | 1.29 | 501.2 | 16.03 | 37.07 | 64.67 | 37.77 | 125.55 |
| 50~52 | 7.89 | 15.90 | 1.38 | 1.31 | 519.4 | 15.13 | 31.67 | 39.02 | 31.47 | 111.45 |
| 52~54 | 8.12 | 15.15 | 1.04 | 1.34 | 517.5 | 15.43 | 35.42 | 44.72 | 33.27 | 162.00 |
| 54~56 | 8.31 | 19.65 | 0.88 | 1.27 | 553.9 | 16.33 | 40.22 | 41.57 | 36.12 | 133.80 |
| 56~58 | 8.70 | 17.25 | 1.48 | 1.39 | 544.7 | 16.18 | 34.82 | 41.42 | 34.17 | 132.60 |
| 58~60 | 8.27 | 18.45 | 1.33 | 1.29 | 541.6 | 16.18 | 35.57 | 41.12 | 37.47 | 12.90 |
| 60~62 | 7.74 | 18.30 | 1.11 | 1.24 | 559.6 | 16.78 | 37.52 | 44.42 | 31.92 | 138.45 |
| 62~64 | 9.28 | 17.70 | 1.40 | 1.42 | 438.6 | 16.03 | 41.27 | 43.67 | 39.57 | 178.50 |
| 64~66 | 7.68 | 18.15 | 1.17 | 1.25 | 534.7 | 16.18 | 40.67 | 43.07 | 38.82 | 157.50 |
| 66~68 | 8.18 | 19.65 | 0.98 | 1.18 | 566.5 | 18.28 | 44.57 | 48.47 | 44.22 | 162.00 |
| 68~70 | 7.35 | 20.40 | 1.22 | 1.10 | 579.2 | 17.83 | 43.67 | 42.77 | 40.17 | 121.20 |
| 70~72 | 7.89 | 18.75 | 1.16 | 1.35 | 518.90 | 17.98 | 42.47 | 45.32 | 39.27 | 130.35 |
| 72~74 | 7.11 | 14.15 | 1.39 | 1.17 | 498.85 | 16.78 | 33.47 | 39.32 | 32.07 | 109.95 |
| 74~76 | 7.57 | 18.60 | 1.26 | 1.20 | 548.04 | 15.73 | 44.27 | 42.32 | 34.92 | 117.15 |
| 76~78 | 8.20 | 20.70 | 1.36 | 1.43 | 516.51 | 16.93 | 40.07 | 40.07 | 35.67 | 119.25 |
