# Supplementary material for: External-forcing modulation on temporal variations of hydrothermalism-evidence from sediment cores in a submarine venting field off northeastern Taiwan
Source: PLoS One. 2018 Nov 29;13(11):e0207774. doi: 10.1371/journal.pone.0207774 (PMC6264505; doi:10.1371/journal.pone.0207774)
Supplement: S3 Table — (DOCX) [file pone.0207774.s003.docx]

**S3 Table. Concentrations of various metals in Core S2 Sediments**

| Depth | Al | Ca | Mg | Mn | Co | Cu | Ni | Pb | Zn |
| --- | --- | --- | --- | --- | --- | --- | --- | --- | --- |
| (cm) | % | % | % | μg g^-1^ | μg g^-1^ | μg g^-1^ | μg g^-1^ | μg g^-1^ | μg g^-1^ |
| 0~2 | 6.76 | 0.97 | 1.16 | 318.4 | 16.19 | 22.60 | 35.64 | 15.48 | 101.82 |
| 2~4 | 6.31 | 1.00 | 1.05 | 310.6 | 16.29 | 23.73 | 37.02 | 15.80 | 97.37 |
| 4~6 | 6.68 | 0.93 | 1.09 | 321.7 | 17.37 | 27.09 | 40.18 | 18.25 | 109.95 |
| 6~8 | 6.40 | 0.95 | 1.09 | 314.4 | 17.45 | 27.69 | 40.57 | 19.01 | 107.19 |
| 8~10 | 7.89 | 1.01 | 1.29 | 368.1 | 17.58 | 29.86 | 41.01 | 19.23 | 114.62 |
| 10~12 | 5.89 | 0.85 | 0.78 | 318.7 | 16.40 | 27.51 | 37.24 | 18.39 | 103.73 |
| 12~14 | 6.49 | 0.78 | 0.91 | 339.6 | 17.30 | 29.77 | 39.36 | 20.08 | 107.82 |
| 14~16 | 5.80 | 0.62 | 0.74 | 320.0 | 17.51 | 31.84 | 40.95 | 21.16 | 114.25 |
| 16~18 | 5.95 | 0.79 | 0.89 | 328.4 | 17.11 | 30.21 | 39.21 | 20.42 | 108.97 |
| 18~20 | 7.37 | 1.09 | 1.23 | 343.3 | 17.16 | 30.42 | 39.60 | 19.33 | 112.72 |
| 20~22 | 6.98 | 1.05 | 1.15 | 341.5 | 17.22 | 30.15 | 41.05 | 20.87 | 111.21 |
| 22~24 | 7.01 | 1.07 | 1.16 | 339.4 | 17.94 | 31.86 | 42.01 | 21.72 | 118.00 |
| 24~26 | 7.18 | 0.63 | 0.84 | 390.8 | 19.50 | 37.91 | 46.67 | 24.08 | 129.25 |
| 26~28 | 7.07 | 1.01 | 1.23 | 339.5 | 18.56 | 32.85 | 41.40 | 21.54 | 116.84 |
| 28~30 | 6.86 | 0.96 | 1.16 | 314.7 | 18.24 | 32.56 | 39.87 | 24.65 | 121.81 |
| 30~32 | 7.05 | 0.83 | 1.18 | 299.7 | 17.57 | 32.06 | 40.39 | 23.67 | 119.85 |
| 32~34 | 6.66 | 0.82 | 1.08 | 307.4 | 17.92 | 32.82 | 39.65 | 23.28 | 114.13 |
| 34~36 | 6.76 | 0.81 | 1.13 | 310.8 | 18.13 | 31.97 | 40.87 | 24.42 | 115.90 |
| 36~38 | 7.95 | 0.93 | 1.28 | 343.1 | 19.31 | 32.16 | 43.00 | 22.92 | 130.01 |
| 38~40 | 6.81 | 1.01 | 1.15 | 339.0 | 19.13 | 30.47 | 41.39 | 22.04 | 120.50 |
| 40~42 | 5.80 | 0.56 | 0.61 | 331.3 | 20.13 | 31.06 | 44.79 | 22.90 | 122.72 |
| 42~44 | 6.54 | 0.64 | 0.81 | 323.4 | 17.36 | 33.97 | 40.28 | 21.57 | 112.82 |
| 44~46 | 7.13 | 0.86 | 1.08 | 354.7 | 17.66 | 35.50 | 40.73 | 21.73 | 118.74 |
| 46~48 | 6.42 | 0.78 | 0.85 | 333.3 | 17.79 | 35.33 | 40.66 | 20.48 | 118.53 |
| 48~50 | 7.49 | 0.99 | 1.15 | 378.5 | 16.99 | 34.20 | 39.90 | 21.16 | 116.24 |
| 50~52 | 7.74 | 1.16 | 1.20 | 402.9 | 16.78 | 38.66 | 38.71 | 21.23 | 116.02 |
| 52~54 | 6.37 | 0.48 | 0.60 | 356.7 | 17.84 | 33.96 | 40.76 | 23.53 | 119.05 |
| 54~56 | 7.35 | 0.86 | 1.09 | 345.4 | 17.86 | 34.11 | 40.03 | 22.68 | 119.65 |
| 56~58 | 6.23 | 1.29 | 1.05 | 365.3 | 16.09 | 32.94 | 38.26 | 22.97 | 123.62 |
| 58~60 | 5.37 | 0.71 | 0.66 | 349.5 | 19.70 | 33.17 | 45.76 | 21.12 | 107.48 |
| 60~62 | 6.17 | 0.93 | 0.85 | 348.0 | 18.27 | 30.79 | 42.86 | 20.77 | 119.68 |
| 62~64 | 6.64 | 1.12 | 1.01 | 372.1 | 18.70 | 33.59 | 42.91 | 21.71 | 123.89 |
| 64~66 | 6.72 | 1.39 | 1.25 | 353.4 | 17.83 | 33.12 | 41.74 | 19.46 | 118.53 |
| 66~68 | 5.20 | 0.78 | 0.67 | 317.3 | 18.95 | 30.94 | 44.29 | 19.52 | 125.20 |
| 68~70 | 7.11 | 1.20 | 1.24 | 342.6 | 17.72 | 34.57 | 41.75 | 18.30 | 117.13 |
| 70~72 | 6.92 | 1.27 | 1.26 | 340.1 | 17.47 | 29.80 | 40.40 | 18.77 | 112.25 |
| 72~74 | 4.84 | 0.69 | 0.65 | 291.9 | 18.86 | 30.08 | 43.59 | 18.40 | 98.56 |
| 74~76 | 6.45 | 0.82 | 0.89 | 326.2 | 15.75 | 30.48 | 35.73 | 18.08 | 120.15 |
| 76~78 | 3.04 | 0.29 | 0.28 | 238.7 | 16.19 | 31.25 | 35.55 | 21.71 | 100.95 |
| 78~80 | 5.11 | 0.50 | 0.53 | 275.0 | 15.61 | 29.87 | 33.69 | 18.90 | 95.27 |
| 80~82 | 5.42 | 0.59 | 0.58 | 309.0 | 16.08 | 31.19 | 35.32 | 19.63 | 98.45 |
| 82~84 | 5.46 | 0.64 | 0.70 | 313.0 | 15.56 | 28.80 | 33.28 | 18.06 | 93.06 |
| 84~86 | 6.81 | 1.34 | 1.37 | 334.45 | 15.66 | 26.72 | 33.01 | 15.78 | 91.51 |
| 86~88 | 6.83 | 1.13 | 1.14 | 339.1 | 15.56 | 28.27 | 33.77 | 16.88 | 95.78 |
| 88~90 | 6.90 | 1.13 | 1.15 | 333.8 | 15.22 | 25.75 | 33.99 | 16.71 | 97.22 |
