# Supplementary material for: External-forcing modulation on temporal variations of hydrothermalism-evidence from sediment cores in a submarine venting field off northeastern Taiwan
Source: PLoS One. 2018 Nov 29;13(11):e0207774. doi: 10.1371/journal.pone.0207774 (PMC6264505; doi:10.1371/journal.pone.0207774)
Supplement: S1 Fig — (DOCX) [file pone.0207774.s004.docx]

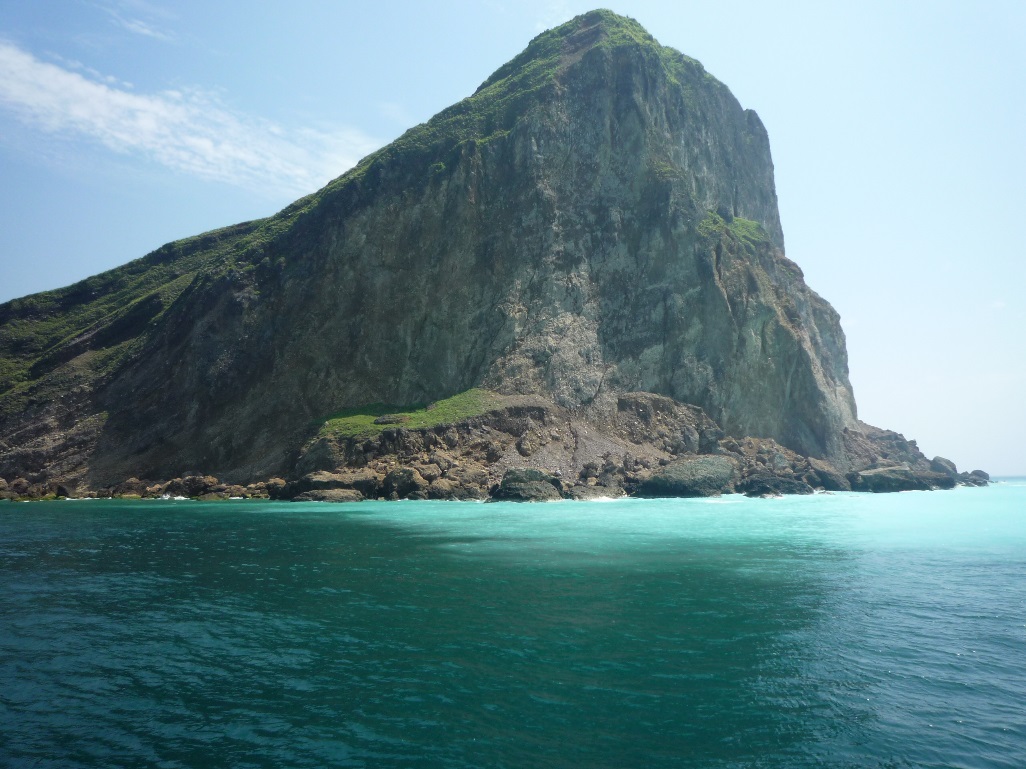


Photo on May 16, 2012

Photo on May 12, 2012


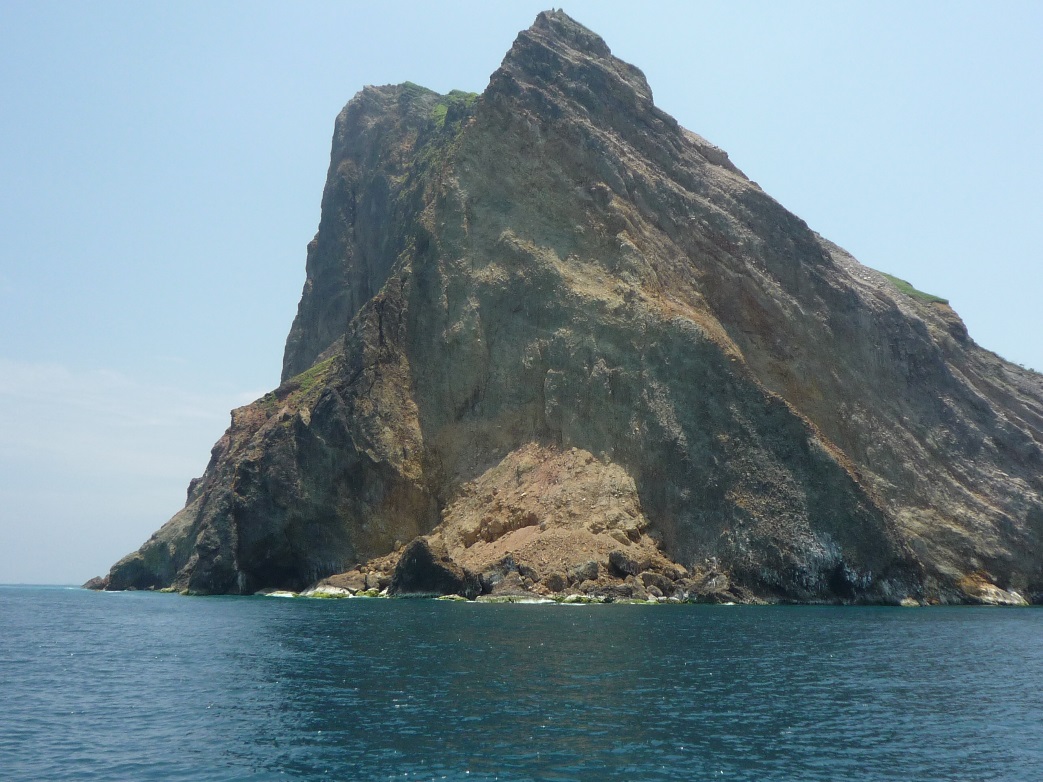


**S1 Fig. Comparison of sulfide spreading zone between 2012 and 2016 on the surface water off the eastern head of Kueishantao Islet.**
